# Effect of Wearing Glasses on Risk of Infection With SARS-CoV-2 in the Community: A Randomized Clinical Trial

**DOI:** 10.1001/jamanetworkopen.2022.44495

**Published:** 2022-12-01

**Authors:** Atle Fretheim, Ingeborg Hess Elgersma, Arnfinn Helleve, Petter Elstrøm, Oliver Kacelnik, Lars G. Hemkens

**Affiliations:** 1 Centre for Epidemic Interventions Research, Norwegian Institute of Public Health, Oslo, Norway; 2 Faculty of Health Sciences, Oslo Metropolitan University, Oslo, Norway; 3 Centre for Evaluation of Public Health Measures, Norwegian Institute of Public Health, Oslo, Norway; 4 Division for Infection Control, Norwegian Institute of Public Health, Oslo, Norway; 5 Department of Clinical Research, University Hospital Basel, University of Basel, Basel, Switzerland; 6 Meta-Research Innovation Center at Stanford (METRICS), Stanford University, Stanford, California; 7 Meta-Research Innovation Center Berlin (METRIC-B), Berlin Institute of Health, Berlin, Germany

## Abstract

**Importance:**

Observational studies have reported an association between the use of eye protection and reduced risk of infection with SARS-CoV-2 and other respiratory viruses, but, as with most infection control measures, no randomized clinical trials have been conducted.

**Objectives:**

To evaluate the effectiveness of wearing glasses in public as protection against being infected with SARS-CoV-2 and other respiratory viruses.

**Design, Setting, and Participants:**

A randomized clinical trial was conducted in Norway from February 2 to April 24, 2022; all adult members of the public who did not regularly wear glasses, had no symptoms of COVID-19, and did not have COVID-19 in the last 6 weeks were eligible.

**Intervention:**

Wearing glasses (eg, sunglasses) when close to others in public spaces for 2 weeks.

**Main Outcomes and Measures:**

The primary outcome was a positive COVID-19 test result reported to the Norwegian Surveillance System for Communicable Diseases. Secondary outcomes included a positive COVID-19 test result and respiratory infection based on self-report. All analyses adhered to the intention-to-treat principle.

**Results:**

A total of 3717 adults (2439 women [65.6%]; mean [SD] age, 46.9 [15.1] years) were randomized. All were identified and followed up in the registries, and 3231 (86.9%) responded to the end of study questionnaire. The proportions with a reported positive COVID-19 test result in the national registry were 3.7% (68 of 1852) in the intervention group and 3.5% (65 of 1865) in the control group (absolute risk difference, 0.2%; 95% CI, −1.0% to 1.4%; relative risk, 1.10; 95% CI, 0.75-1.50). The proportions with a positive COVID-19 test result based on self-report were 9.6% (177 of 1852) in the intervention group and 11.5% (214 of 1865) in the control group (absolute risk difference, –1.9%; 95% CI, −3.9% to 0.1%; relative risk, 0.83; 95% CI, 0.69-1.00). The risk of respiratory infections based on self-reported symptoms was lower in the intervention group (30.8% [571 of 1852]) than in the control group (34.1% [636 of 1865]; absolute risk difference, –3.3%; 95% CI, −6.3% to −0.3%; relative risk, 0.90; 95% CI, 0.82-0.99).

**Conclusions and Relevance:**

In this randomized clinical trial, wearing glasses in the community was not protective regarding the primary outcome of a reported positive COVID-19 test. However, results were limited by a small sample size and other issues. Glasses may be worth considering as one component in infection control, pending further studies.

**Trial Registration:**

ClinicalTrials.gov Identifier: NCT05217797

## Key Points

**Question:**

What is the effect of wearing glasses on the risk of being infected with SARS-CoV-2 and other respiratory viruses?

**Findings:**

In this randomized clinical trial with 3717 participants, there was no statistically significant difference in the incidence of positive reported COVID-19 cases. There was a statistically significant lower self-reported incidence of respiratory infection in the intervention group.

**Meaning:**

This study does not conclude that recommending the use of glasses to prevent infection with SARS-CoV-2 and other respiratory viruses is beneficial, but the intervention is worth considering because it is simple, low cost, and has few negative consequences.

## Teaser

This randomized clinical trial evaluates the effectiveness of wearing glasses in public as protection against being infected with SARS-CoV-2 and other respiratory viruses.

## Introduction

Use of eye protective gear for infection control was proposed more than 100 years ago^1^ but has received little attention during the ongoing COVID-19 pandemic, except as part of personal protective equipment for health care workers.^2^ A simple means of eye protection is to wear glasses. Many people have easy access to sunglasses, and it requires little effort to use them in everyday life. Repurposing sunglasses for infection control could be a simple, safe, and environmentally friendly infection prevention measure. An association between wearing glasses and lower risk of respiratory viruses has been reported from observational studies, but, to our knowledge, no randomized clinical trial of eye protection for respiratory virus infection has been conducted, either in health care or community settings.^3,4,5,6^ To inform decisions about the use of personal protection against SARS-CoV-2, we carried out a randomized clinical trial of the effect of wearing glasses on the risk of being infected with SARS-CoV-2 and other respiratory viruses.

## Methods

### Participants

This pragmatic randomized clinical trial was conducted in Norway from February 2 to April 24, 2022. The trial was pragmatic in the sense that it aimed at informing decisions about using glasses for infection control by determining their effectiveness in real life.^7^ We recruited participants via an online portal distributed to the Norwegian public through the media, online and print advertisements, and e-mails to members of survey panels of 2 data collection companies. All members of the public could take part if they (1) were at least 18 years of age; (2) did not regularly wear glasses; (3) owned or could borrow glasses that they could use (eg, sunglasses); (4) had not contracted COVID-19 in the 6 weeks prior to participation; (5) did not have COVID-19 symptoms when providing consent; (6) were willing to be randomly assigned to wear or not wear glasses outside their home when close to others for a 2-week period; and (6) provided informed consent. Persons dependent on visual aids who could use contact lenses if allocated to the control group were eligible.

Information about the goal of the trial and eligibility study were provided through the online portal, and eligible participants consented to participation using the national digital signature service (Posten signering). In our judgment, taking part entailed negligible risk, and the findings can potentially inform decisions about infection control measures in the ongoing and future epidemics. Thus, we considered the risk-benefit ratio morally defensible. The protocol was approved by the Regional Ethics Committee of South-East Norway. There were no major deviations from the protocol (trial protocol in Supplement 1). We followed the Consolidated Standards of Reporting Trials (CONSORT) reporting guideline extension for pragmatic trials.^7^ Neither patients nor the public were involved in the design, conduct, reporting, or dissemination plans of this study. The trial is registered at ClinicalTrials.gov (NCT05217797).

### Intervention

The participants randomly assigned to the intervention group were asked to wear sunglasses or other types of glasses when close to other people outside their home (on public transportation, in shopping malls, etc), for a 14-day period. The control group was encouraged not to wear glasses when close to others outside their home.

### Outcomes

All data were collected from national registries or at the end of study survey. In general, data from administrative registries in Norway are reliable and of high quality and are used extensively in research.^8^ All participants had to identify themselves using their personal identification number and were thereby identifiable in the national health registries. All polymerase chain reaction (PCR) COVID-19 tests are analyzed by laboratories that report results directly to the Norwegian Surveillance System for Communicable Diseases (MSIS), so we can reasonably assume that all such tests are registered. We used routinely collected data and did not set up our own system for COVID-19 testing. We assumed that the participants would undergo testing for COVID-19 if they had relevant symptoms, in line with recommendations from the health authorities.

The primary outcome was a positive COVID-19 test result reported to the MSIS (days 3-17 of the study period). Secondary outcomes included (1) a positive COVID-19 test result (self-reported; days 1-17 of study period); (2) episode of respiratory infection (self-reported symptoms; days 1-17 of study period), defined as having 1 respiratory symptom (stuffed or runny nose, sore throat, cough, sneezing, or heavy breathing) and fever or 1 respiratory symptom and at least 2 more symptoms (body ache, muscular pain, fatigue, reduced appetite, stomach pain, headache, and/or loss of smell); (3) health care use for respiratory symptoms (self-reported; days 1-17); (4) health care use for injuries (self-reported; days 1-17); (5) health care use, all causes (self-reported; days 1-17); (6) health care use for respiratory symptoms (registered in the Norwegian Patient Registry [NPR]; days 3-28); (7) health care use for injuries (registered in the NPR and the Norwegian Registry for Primary Health Care [KPR]; days 1-21); and (8) health care use, all causes (registered in the NPR and KPR; days 1-21). We do not report on the latter 4 outcomes here because we do not have access to the data for the time being.

We also asked the participants about adherence to the intervention, use of face masks, testing behavior, and whether they had any negative experiences with taking part in the study (questionnaire in the eAppendix in Supplement 2). For the COVID-19 outcome based on registry data, we did not consider events reported in the first 2 days after randomization, to reflect the asymptomatic early infection period. For the other COVID-19 outcomes, we avoided relying on the exact recall of the date of infection and considered the complete follow-up period.

### Sample Size

We originally estimated a need for approximately 22 000 participants, based on an expected event rate of 2% in the control group and 1.5% in the intervention group. The underlying assumptions are provided in the trial protocol in Supplement 1.

### Randomization and Blinding

The participants were automatically randomized and informed about group allocation immediately after signing the consent form in the online recruitment platform (Nettskjema). No blinding was feasible, except for in the analysis phase. One member of our team (A.H.) provided the chief analyst (I.H.E.) with a data file in which group allocation was blinded. The chief analyst presented the main results to the project team members, who were also blinded to the group allocation (and data on use of glasses). We discussed how we would interpret the findings depending on whether one group or the other was the intervention group, and we prepared a short report that we posted online before unblinding ourselves to the group allocation.^9^

### Data Management

We used the University of Oslo’s web-based survey platform Nettskjema for screening and data collection, and we used their service for secure storage of research data (Service for Sensitive Data [TSD]). We collected directly identifiable data (name, personal identification number, and email address). Along with a code for linking data, the personal identification number was sent to the registries. Each register deleted the personal identification number before the registry data and code for linking were delivered and stored in TSD. The questionnaire data were processed in the same way. The codes for linking data to the personal identification number were kept in a securely stored database, with limited access.

### Statistical Analysis

We conducted unadjusted analyses to estimate the relative risk (RR) and risk difference for the prespecified outcomes, with 95% CIs calculated with the Wald method. All analyses adhered to the intention-to-treat principle, and we included all randomized participants in the denominator in all our main analyses. When outcome data were missing, we assumed that there was no event. As a post hoc sensitivity analysis, we also conducted a crude (unadjusted) per-protocol analysis in which we included only those in the intervention group who reported wearing glasses more than 50% of the time and those in the control group who reported wearing glasses less than 50% of the time.

As planned, we conducted a subgroup analysis for participants who used contact lenses, hypothesizing smaller effects because control group participants were allowed to use contact lenses. One possible mechanism is that the viruses attach to angiotensin-converting enzyme 2 receptors in the cornea, which we assume are protected by contact lenses.^5^ We also conducted subgroup analyses for vaccination status and history of COVID-19 using a χ^2^ test of interaction. All *P* values were from 2-sided tests and results were deemed statistically significant at *P* < .05.

## Results

**Figure 1.**
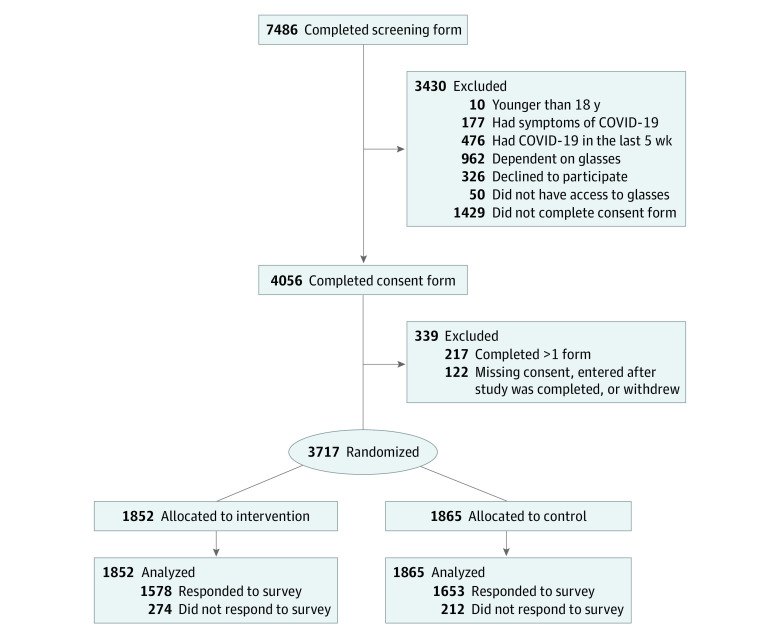
Flow Diagram of Participants

**Figure 2.**
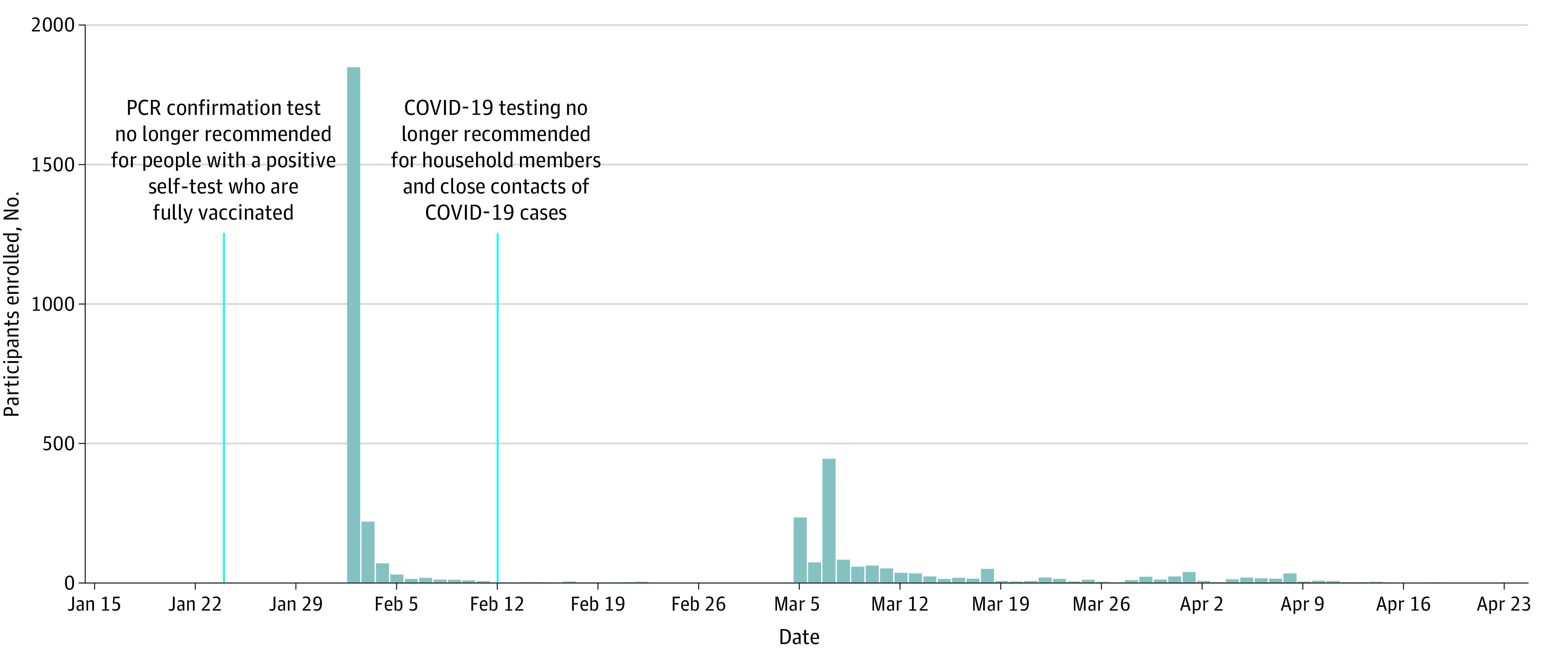
Recruitment Timeline Gray bars indicate participants enrolled each day. Blue lines indicate changes in testing recommendations. PCR indicates polymerase chain reaction.

Of the 7506 individuals who completed the screening form, 3717 (49.5%; mean [SD] age, 46.9 [15.1] years; 2439 [65.6%] women; 2873 [77.3%] with ≥3 doses of COVID-19 vaccine) were randomized (Table 1; Figure 1). Recruitment started on February 2, 2022, and closed April 24, 2022. Most participants entered the trial soon after initiation, after wide media coverage on Norwegian national television and other media outlets (Figure 2). Another wave of recruitment occurred after 2 data collection companies invited members of their survey panels to take part in the trial.

**Table 1.** Baseline Characteristics of Trial Participants

Characteristic	Participants, No. (%)	
	Intervention group (n = 1852)	Control group (n = 1865)
Sex		
Female	1193 (64.4)	1246 (66.8)
Male	659 (35.6)	619 (33.2)
Age, mean (SD), y	46.9 (15.1)	46.8 (15.1)
No. of COVID-19 vaccine doses received		
0	50 (2.7)	58 (3.1)
1	19 (1.0)	21 (1.1)
2	361 (19.5)	335 (18.0)
≥3	1422 (76.8)	1451 (77.8)

The main findings are summarized in Table 2. For reported COVID-19 cases, the proportions were 68 of 1852 (3.7%) in the intervention group and 65 of 1865 (3.5%) in the control group (absolute risk difference, 0.2%; 95% CI, −1.0% to 1.4%; RR, 1.10; 95% CI, 0.75-1.50). The proportion with self-reported positive test results was lower in the intervention group, but the result was not statistically significant (177 of 1852 [9.6%]) than in the control group (214 of 1865 [11.5%]; absolute risk difference, –1.9%; 95% CI, −3.9% to 0.1%; RR, 0.83; 95% CI, 0.69-1.00). Almost one-third of all participants reported symptoms of respiratory infection, with a lower proportion in the intervention group (571 of 1852 [30.8%]) than in the control group (636 of 1865 [34.1%]; absolute risk difference, –3.3%; 95% CI, −6.3% to −0.3%; RR, 0.90; 95% CI, 0.82-0.99). There were 11 reported COVID-19 cases among the nonrespondents to the survey (8 in the intervention group and 3 in the control group).

**Table 2.** Main Findings

Finding	Participants, No. (%)		Relative risk (95% CI)	Absolute risk difference, % (95% CI)
	Intervention group (n = 1852)	Control group (n = 1865)		
Reported COVID-19 case^a^	68 (3.7)	65 (3.5)	1.10 (0.75-1.50)	0.2 (−1.0 to 1.4)
Self-reported COVID-19 case^b^	177 (9.6)	214 (11.5)	0.83 (0.69-1.00)	−1.9 (−3.9 to 0.1)
Self-reported respiratory infection	571 (30.8)	636 (34.1)	0.90 (0.82-0.99)	−3.3 (−6.3 to −0.3)
Health care use, all cause	80 (4.3)	83 (4.5)	0.97 (0.72-1.30)	−0.1 (−1.4 to 1.2)
Self-reported health care use due to airway symptoms	15 (0.8)	20 (1.1)	0.76 (0.39-1.50)	−0.3 (−0.9 to 0.4)
Self-reported health care use due to injuries	24 (1.3)	16 (0.9)	1.50 (0.81-2.80)	0.4 (−0.2 to 1.1)

^a^ Date of test between day 3 and day 17 after inclusion in the study.

^b^ Date of test between day 1 and day 17 after inclusion in the study.

We report behavioral outcomes in Table 3. In the intervention group, 70.5% of the participants (1306 of 1852) reported using glasses at least 50% of the time, while the corresponding proportion in the control group was 10.5% ([196 of 1865]; absolute risk difference, 60.0%; 95% CI, 57.5%-62.5%). In the intervention group, 39.5% of the participants (731 of 1852) reported using face masks at least 50% of the time, while the corresponding proportion in the control group was 29.7% ([554 of 1865]; absolute risk difference, 9.8%; 95% CI, 6.7%-12.8%). The proportion of participants who took a COVID-19 test during the trial period was similar in the intervention and control groups (925 of 1852 [49.9%] vs 914 of 1865 [49.0%]). We also asked the participants whether they normally commuted to work or school by public transportation, and the proportion reporting that they did was lower in the intervention group (427 of 1852 [23.1%]) than in the control group (508 of 1865 [27.2%]; absolute risk difference, –4.2%; 95% CI, −7.0% to −1.4%).

**Table 3.** Behavioral Outcomes

Outcome	Participants, No. (%)		Absolute risk difference, % (95% CI)
	Intervention group (n = 1852)	Control group (n = 1865)	
Responded to survey	1578 (85.2)	1653 (88.6)	−3.4 (−5.6 to −1.3)
**Use of glasses**			
At least 50% of the time	1306 (70.5)	196 (10.5)	60.0 (57.5 to 62.5)
Always	575 (31.0)	60 (3.2)	27.8 (25.6 to 30.1)
Almost always (75%-100% of the time)	524 (28.3)	81 (4.3)	24.0 (21.7 to 26.2)
Often (50%-75% of the time)	207 (11.2)	55 (2.9)	8.2 (6.6 to 9.9)
Sometimes (25%-50% of the time)	91 (4.9)	59 (3.2)	1.8 (0.5 to 3.0)
Almost never (≤25% of the time)	101 (5.5)	339 (18.2)	−12.7 (−14.8 to −10.7)
Never	78 (4.2)	1054 (56.5)	−52.3 (−54.7 to −49.9)
**Use of face masks**			
At least 50% of the time	731 (39.5)	554 (29.7)	9.8 (6.7 to 12.8)
Always	180 (9.7)	134 (7.2)	2.5 (0.7 to 4.3)
Almost always (75%-100% of the time)	319 (17.2)	225 (12.1)	5.2 (2.9 to 7.4)
Often (50%-75% of the time)	232 (12.5)	195 (10.5)	2.1 (0.0 to 4.1)
Sometimes (25%-50% of the time)	210 (11.3)	227 (12.2)	−0.8 (−2.9 to 1.2)
Almost never (≤25% of the time)	250 (13.5)	345 (18.5)	−5.0 (−7.4 to −2.6)
Never	381 (20.6)	524 (28.1)	−7.5 (−10.3 to −4.8)
**Use of COVID-19 tests**			
Tested	925 (49.9)	914 (49.0)	0.9 (−2.3 to 4.2)
Home test and test station	120 (6.5)	103 (5.5)	1.0 (−0.6 to 2.5)
Only test station	10 (0.5)	13 (0.7)	−0.2 (−0.7 to 0.3)
Only home test	795 (42.9)	798 (42.8)	0.1 (−3.0 to 3.3)
No	651 (35.2)	739 (39.6)	−4.5 (−7.6 to −1.4)

The results of the per-protocol analysis are presented in eTable 1 in Supplement 2. There were practically no differences between these estimates and those of the main analysis. Subgroup analyses did not suggest any interaction effects, except between vaccination status and relative risk of reported COVID-19 (eTables 2 and 3 in Supplement 2).

In total, 76 participants reported having negative experiences with taking part in the trial (53 in the intervention group and 23 in the control group), and all but 1 elaborated on these experiences using free text. The most common experience concerned the combination of glasses and face masks, especially fogging of glasses (21 in the intervention group). Wearing glasses felt uncomfortable or tiring to some participants, and a few participants reported reduced vision because of using sunglasses or reading glasses. One person in the intervention group reported a fall because of reduced vision. A few reported feeling silly when wearing glasses or sunglasses (eg, inside a café). In the control group, some participants reported headaches or other problems from not being able to use glasses.

## Discussion

The findings from this first randomized clinical trial of eye protection against viral spread are inconclusive but indicate that wearing glasses in public may protect against respiratory viruses. The evidence is uncertain, and more trials are needed to corroborate this evidence.

### Comparison With Other Studies

A recent systematic review of studies estimating the association of eye protection with transmission of SARS-CoV-2 identified 5 observational studies, all conducted among health care workers in various settings.^5^ With 1 exception, all studies reported a clear association between the use of eye protection and reduced infection risk, with estimated RR reductions ranging from 40% to 96%. The authors also identified 3 cross-sectional studies examining the use of glasses in the community, all of which found a substantial underrepresentation of people wearing glasses among patients with COVID-19.^10,11,12^ In addition, some studies have shown high detection rates of SARS-CoV-2 in ocular fluids among patients with COVID-19, also suggesting that the eyes may be an important route of viral transmission.^13,14^ A systematic review from 2020 of studies from previous coronavirus outbreaks (MERS [Middle East respiratory syndrome] and SARS) included 12 studies in a meta-analysis indicating that use of eye protection was associated with considerable reduced infection risk (RR, 0.34; 95% CI, 0.21-0.56).^4^ None of the studies were randomized, and nearly all were conducted in health care settings. Furthermore, a Cochrane review from 2020 identified no randomized clinical trials of eye protection to curb the spread of respiratory viruses.^3^ While we were conducting our trial, a preprint with findings from a cohort study in the UK was published, reporting 15% lower odds of COVID-19 among consistent users of glasses (odds ratio, 0.85; 95% CI, 0.77-0.94), after adjusting for key variables (eg, age).^15^ Overall, these prior findings are compatible with the results of our trial. We were able to perform this trial with relative ease, so we are hopeful that others will conduct similar trials that can be synthesized in meta-analyses and provide a more robust body of evidence.^16^

To our knowledge, our trial is one of very few randomized clinical trials evaluating public health and social measures for epidemic control.^3,4,6^ For example, only 2 randomized clinical trials of face masks have been conducted during the COVID-19 pandemic.^17,18^ The results from the 2 face mask trials are, broadly speaking, in the same range as ours (ie, consistent with a modest, albeit uncertain, effect).

### Limitations and Strengths

This study has some limitations. The result for our primary end point is inconclusive, with a wide 95% CI compatible with both important benefit and harm. We would probably have achieved a more precise estimate had we been more successful with our recruitment of participants. We decided to end the trial after 12 weeks given the decreasing incidence of COVID-19 and because recruitment was becoming increasingly difficult. Despite wide media coverage, we came nowhere near our target number of participants. Also, 1 week before we opened recruitment into the trial, the health authorities changed their recommendations for testing, no longer advising a confirmatory PCR test for fully vaccinated people who self-tested positive for COVID-19. Then, 10 days after we started recruitment, the authorities changed their testing recommendations again, revoking the earlier advice that members of households with a COVID-19 case should get tested, and recommended that only adults with symptoms should test themselves. These policy changes were followed by a drastic reduction in the number of test results reported to the MSIS. We therefore considered changing our main outcome from reported COVID-19 cases to self-reported cases but decided to adhere to the protocol. Consequently, we chose to also put some emphasis on the secondary outcomes in our interpretation of the study findings. As a safeguard, we assessed the results while being blinded to group allocation, and we prespecified how we would interpret the findings depending on whether one group or the other was the intervention group.^9^

There is a risk of bias for survey-based outcomes because we lacked responses from 486 participants (13.1%). However, very few reported COVID-19 cases were seen among the nonrespondents, indicating a low risk of bias.

This study has some strengths. We believe the pragmatic trial approach we adopted is particularly appropriate for evaluations of public health interventions in which clinical effectiveness is of key interest.^19,20,21^ However, we were not able to clearly assess to what extent the observed effects were due to increased wearing of glasses, increased use of face masks, differences in the use of public transportation, decreased awareness of symptoms, or decreased reporting of outcomes. Consistent with the pragmatic intent, we sought to be as close as possible to the natural environment, with minimal interaction with participants. However, our short questionnaire at the end of the study provided specific information to better understand how the intervention worked, indicating that the intervention did increase the use of eye protection, which then contributed to possible reduced transmission of respiratory infections.

A surprise finding to us is that the use of face masks was substantially higher in the intervention group, especially because difficulties with combining glasses and face masks was the most common negative experience reported. Although we believe that our findings are likely to be applicable in many settings beyond ours, there are some factors that may reduce the applicability. We conducted our trial during a phase of the pandemic when the Omicron variant of the virus dominated. It is possible that new variants, or other respiratory viruses, have different modes of transmission. The trial took place during the Norwegian winter, and the importance of transmission in public spaces may be different in other seasons or cultural settings. The COVID-19 incidence was exceptionally high during the time of the trial. Also, the effects that the intervention had on behavior (eg, face mask use) will likely vary across contexts and populations.

## Conclusions

In this randomized clinical trial, wearing glasses in the community did not have a protective effect regarding the primary outcome of a reported positive COVID-19 test result. However, study results were limited by small sample sizes and other issues, rendering the findings inconclusive as to whether recommending the use of glasses in public reduces the risk of respiratory infections. Any effect would probably be modest at best, but wearing glasses is simple, low burden, and low cost; has few negative consequences; and may be worth considering as 1 component in infection control, pending further studies.

## Notes

Supplement 1.
Trial Protocol

Supplement 2. **eAppendix.** Questionnaire (Translated From Norwegian) eTable 1. Crude Per Protocol Analysis, ie, Only Including Those in the Intervention Group Who Reported Wearing Glasses More Than 50% of the Time, and Those in the Control Group Who Reported Wearing Glasses Less Than 50% of the Time eTable 2. Subgroup Analysis Using Notified COVID-19 Cases as Outcome eTable 3. Subgroup Analysis for Using Self-Reported COVID-19 Cases as Outcome

Supplement 3.
Data Sharing Statement
