# Supplementary material for: Effect of Wearing Glasses on Risk of Infection With SARS-CoV-2 in the Community: A Randomized Clinical Trial
Source: JAMA Netw Open. 2022 Dec 1;5(12):e2244495. doi: 10.1001/jamanetworkopen.2022.44495 (PMC9716386; doi:10.1001/jamanetworkopen.2022.44495)
Supplement: Supplement 2. — eAppendix. Questionnaire (Translated From Norwegian) eTable 1. Crude Per Protocol Analysis, ie, Only Including Those in the Intervention Group Who Reported Wearing Glasses More Than 50% of the Time, and Those in the Control Group Who Reported Wearing Glasses Less Than 50% of the Time eTable 2. Subgroup Analysis Using Notified COVID-19 Cases as Outcome eTable 3. Subgroup Analysis for Using Self-Reported COVID-19 Cases as Outcome [file jamanetwopen-e2244495-s002.pdf]

Supplementary Online Content

Fretheim A, Elgersma IH, Helleve A, Elstrøm P, Kacelnik O, Hemkens LG. Effect of wearing glasses on risk of infection with SARS-CoV-2 in the community: a randomized clinical trial. *JAMA Netw Open.* 2022;5(12):e2244495.
doi:10.1001/jamanetworkopen.2022.44495

eAppendix. Questionnaire (Translated From Norwegian)
eTable 1. Crude Per Protocol Analysis, ie, Only Including Those in the Intervention Group Who Reported Wearing Glasses More Than 50% of the Time, and Those in the Control Group Who Reported Wearing Glasses Less Than 50% of the Time
eTable 2. Subgroup Analysis Using Notified COVID-19 Cases as Outcome
eTable 3. Subgroup Analysis for Using Self-Reported COVID-19 Cases as Outcome

This supplementary material has been provided by the authors to give readers additional information about their work.

© 2022 Fretheim A et al. *JAMA Network Open.*

eAppendix. Questionnaire (Translated From Norwegian)

Questions	Type of response	Options
1. Do you wear contact lenses?	Categories	Yes, /No
2. How often over the last two weeks have you been using glasses (reading glasses, sunglasses, sports glasses etc.) when you have been close to others outside your home?		Always/Almost always (at least 75% of the time)/ Often (50-75% of the time)/Sometimes (25-50 % of the time)/A few times (up to 25% of the time)/Never (0% of the time)
3. How often over the last two weeks have you used a face mask when you have been close to others outside your home?		Always/Almost always (at least 75% of the time)/ Often (50-75% of the time)/Sometimes (25-50 % of the time)/A few times (up to 25% of the time)/Never (0% of the time)
3. Do you normally use public transportation (bus, train, tram, metro, ferry, taxi) to and from work/study place?	Categories	Yes/No
4. Have you experienced one of the following symptoms since taking part in the study?	Categories	Headache/Fever/Clogged nose or runny nose/ Reduced sense of smell/Reduced appetite/ Sore throat /Cough/ Sneezing/Body aches/ Muscle pain/Fatigue, lethargy/Heavy breathing/Abdominal pain
5.Have you taken a COVID-19 over the last two weeks (self-test/test at testing facility)?	Categories	Yes, both self-test and at testing facility/Yes, but only a test station/Yes, but only self-test/No
7. What was the test result?	Categories	Positive/Negative/Unsure
8. When did you take the test?		Date
9. Have you needed any medical care after you took part in the study?	Categories	Yes/No
Item 10-11 if response “Yes” on item 9		
10. Did you need for medical care due to respiratory symptoms?	Categories	Yes/No
11. Did you need for medical care due to injuries?	Categories	Yes/No
12. Have you had any negative experiences from participating in this study?	Categories	Yes/No
Item 13 if response “Yes” on item 12		
13. Please describe what those experiences were		Free text

© 2022 Fretheim A et al. *JAMA Network Open.*

eTable 1. Crude Per Protocol Analysis, ie, Only Including Those in the Intervention Group Who Reported Wearing Glasses More Than 50% of the Time, and Those in the Control Group Who Reported Wearing Glasses Less Than 50% of the Time

	Not wearing glasses in control group (n=1507)	Wearing glasses in intervention group (n=1306)	Risk ratio	Absolute risk difference
Notified COVID-19 case[1]	57/1507 (3.8%)	50/1306 (3.8%)	1.00 (0.70 to 1.50)	0.0% (-1.4 to 1.5)
Self-reported COVID-19 case[2]	201/1507 (13.3%)	143/1306 (10.9%)	0.82 (0.67 to 1.00)	-2.4% (-4.8 to 0.0)
Respiratory infection[3]	593/1507 (39.3%)	462/1306 (35.4%)	0.90 (0.82 to 0.99)	-4.0% (-7.6 to -0.4)
Health care use, all cause[3]	77/1507 (5.1%)	69/1306 (5.3%)	1.00 (0.75 to 1.40)	0.2% (-1.5 to 1.8)
Health care use, due to airway symptoms[3]	16/1507 (1.1%)	13/1306 (1.0%)	0.94 (0.45 to 1.90)	-0.1% (-0.8 to 0.7)
Health care use, due to injuries[3]	15/1507 (1.0%)	22/1306 (1.7%)	1.70 (0.88 to 3.20)	0.7% (-0.2 to 1.5)

[1]Between day 3 and day 17 after inclusion in the study
[2]Between day 1 and day 17 after inclusion in the study
[3]Self reported

© 2022 Fretheim A et al. *JAMA Network Open.*

eTable 2. Subgroup Analysis Using Notified COVID-19 Cases as Outcome

Variable	Subgroup	Intervention group	Control group	Relative risk	Absolute risk difference	P-value for interaction
Lenses	Yes	14/333 (4.2%)	20/384 (5.2%)	0.81 (0.41 to 1.60)	-1.0% (-4.1 to 2.1)	0.41
	No	46/1241 (3.7%)	42/1267 (3.3%)	1.10 (0.74 to 1.70)	0.4% (-1.0 to 1.8)	
Vaccine status	0	5/50 (10.0%)	4/58 (6.9%)	1.40 (0.41 to 5.10)	3.1% (-7.5 to 13.7)	0.015
	1	5/19 (26.3%)	0/21 (0.0%)	-	26.3% (6.5 to 46.1)	
	2	23/361 (6.4%)	30/335 (9.0%)	0.71 (0.42 to 1.20)	-2.6% (-6.5 to 1.4)	
	3+	35/1422 (2.5%)	31/1451 (2.1%)	1.20 (0.71 to 1.90)	0.3% (-0.8 to 1.4)	
COVID-19 previously	No	66/1708 (3.9%)	63/1743 (3.6%)	1.10 (0.76 to 1.50)	0.2% (-1.0 to 1.5)	0.82
	Yes	2/144 (1.4%)	2/122 (1.6%)	0.85 (0.12 to 5.90)	-0.3% (-3.2 to 2.7)	

© 2022 Fretheim A et al. *JAMA Network Open.*

eTable 3. Subgroup Analysis for Using Self-Reported COVID-19 Cases as Outcome

Variable	Subgroup	Intervention group	Control group	Relative risk	Absolute risk difference	P-value for interaction
Lenses	Yes	41/333 (12.3%)	65/384 (16.9%)	0.73 (0.51 to 1.00)	-4.6% (-9.8 to 0.5)	0.26
	No	135/1241 (10.9%)	149/1267 (11.8%)	0.93 (0.74 to 1.20)	-0.9% (-3.4 to 1.6)	
Vaccine status	0	6/50 (12.0%)	6/58 (10.3%)	1.20 (0.40 to 3.40)	1.7% (-10.3 to 13.6)	0.15
	1	4/19 (21.1%)	2/21 (9.5%)	2.20 (0.46 to 11.00)	11.5% (-10.7 to 33.7)	
	2	39/361 (10.8%)	60/335 (17.9%)	0.60 (0.41 to 0.88)	-7.1% (-12.3 to -1.9)	
	3+	128/1422 (9.0%)	146/1451 (10.1%)	0.89 (0.71 to 1.10)	-1.1% (-3.2 to 1.1)	
COVID-19 previously	No	175/1708 (10.2%)	211/1743 (12.1%)	0.85 (0.70 to 1.00)	-1.9% (-4.0 to 0.2)	0.65
	Yes	2/144 (1.4%)	3/122 (2.5%)	0.56 (0.096 to 3.30)	-1.1% (-4.4 to 2.3)	

© 2022 Fretheim A et al. *JAMA Network Open.*
