# Supplementary material for: Effect of Wearing Glasses on Risk of Infection With SARS-CoV-2 in the Community: A Randomized Clinical Trial
Source: JAMA Netw Open. 2022 Dec 1;5(12):e2244495. doi: 10.1001/jamanetworkopen.2022.44495 (PMC9716386; doi:10.1001/jamanetworkopen.2022.44495)
Supplement: Supplement 3. — Data Sharing Statement [file jamanetwopen-e2244495-s003.pdf]

Data Sharing Statement

Fretheim. Effect of Wearing Glasses on Risk of Infection With SARS-CoV-2 in the Community. *JAMA Netw Open*. Published December 01, 2022. doi:10.1001/jamanetworkopen.2022.44495

Data
Data available: Yes
Data types: Deidentified participant data
How to access data: Requests for data must be sent to corresponding author, atle.fretheim@fhi.no
When available: With publication

Supporting Documents
Document types: Other (please specify)
Additional Information: Report of blinding assessment of study results.
How to access documents: https://zenodo.org/record/6669688
When available: beginning date: 06-20-2022

Additional Information
Who can access the data: Anyone.
Types of analyses: Any
Mechanisms of data availability: Online.
Any additional restrictions: None
